# Practical exploration of BOPPPS model combined with situational teaching method in clinical training of intensive medicine: novel pedagogy and perception

**DOI:** 10.3389/fmed.2024.1442099

**Published:** 2024-10-18

**Authors:** Yanquan Liu, Xiaolan Lian, Xiaojun Chen, Minjuan Zeng, Yue Yin, Jie Lin

**Affiliations:** ^1^The First School of Clinical Medicine, Guangdong Medical University, Dongguan, Guangdong, China; ^2^Fujian Medical University Union Hospital, Fuzhou, Fujian, China; ^3^The Affiliated Hospital of Putian University, Putian, Fujian, China; ^4^The School of Basic Medicine, Guangdong Medical University, Dongguan, Guangdong, China; ^5^Department of Intensive Medicine (Comprehensive ICU), The First Affiliated Hospital of Gannan Medical University, Ganzhou, Jiangxi, China

**Keywords:** BOPPPS, teaching model, situational teaching method, ICU, clinical training, pedagogy

## Abstract

**Objective:**

To explore the application value of bridge-in, learning objective, pre-assessment, participatory learning, post-assessment, and summary (BOPPPS) model combined with situational teaching method in the clinical teaching of intensive care unit (ICU), and to provide experience for the reform of ICU clinical teaching and standardized training of intensive medicine.

**Methods:**

A randomized trial was conducted using a multi-center, prospective cohort study. A total of 293 residential physicians in ICU of Fujian Medical University Union Hospital, the Affiliated Hospital of Putian University and the First Affiliated Hospital of Gannan Medical University from January 2021 to December 2023 were selected as subjects, and the residential physicians in ICU in each medical center were divided into control group and experimental group using random number table method. The control group adopted bedside teaching and demonstration teaching method, and the experimental group adopted BOPPPS model combined with situational teaching method. Clinical teaching performance was evaluated by ICU admission examination, and study process questionnaire (SPQ) and the critical thinking disposition inventory-Chinese version (CTDI-CV) was used to evaluate the learning motivation and critical thinking ability of the two groups. At the same time, the effect evaluation and satisfaction questionnaire of ICU training (EESQ) was used to evaluate the teaching satisfaction.

**Results:**

The scores of the experimental group in ICU theoretical knowledge, clinical thinking and skills, and the treatment of clinical critical cases were higher than those of the control group [(87.31 ± 13.15), (92.86 ± 12.35), (81.45 ± 11.28)] vs. [(83.94 ± 12.73), (88.37 ± 12.61), (78.83 ± 10.47)], the difference between the two groups was statistically significant (*p* < 0.05). The scores of surface motivation, deep motivation, achievement motivation and SPQ total scores of the experimental group were all higher than those in control group (*p* < 0.05), and the scores of seek truth, open mind, analytical ability, systematic ability, self-confidence of critical thinking and intellectual curiosity of the experimental group were also higher than those in the control group, and the CTDI total score was statistically significant (*p* < 0.05). In addition, the results of the questionnaire showed that the experimental group was better than the control group in terms of learning interest in ICU, improvement of humanistic care and doctor-patient communication ability, improvement of teamwork ability, improvement of job identity, ICU training harvest and satisfaction with teacher style (*p* < 0.05).

**Conclusion:**

The combination of BOPPPS model and situational teaching method is likely a more effective and helpful which may improve the clinical comprehensive ability and training quality of residential physicians, and it may be worth learning and promoting.

## 1 Introduction

Intensive care unit (ICU), as a clinical center of diagnosis and treatment for critical diseases and a platform for intensive medicine in hospitals, is particularly necessary to train qualified ICU residents and improve the clinical comprehensive ability of resident physicians due to its strong professionalism, complex clinical diagnosis and treatment process and extremely heavy daily diagnosis and treatment tasks (1, 2). However, due to the particularity and complexity of daily diagnosis and treatment in the ICU, clinical teaching has become difficult or even burdensome, such as the educational background of trainees, the variability of patients’ conditions in the ICU, time and space constraints, and so on. Teaching in the high-intensity and stressful environment of the ICU is challenging and may limit the growth and improvement of young residents. While it is undeniable that the ICU provides unique educational opportunities for medical undergraduates and graduates, including daily emergency training in critical medicine, the use and management of ventilators, the cultivation of good doctor-patient communication skills, and the teaching and experience sharing of lectures on dynamic topics of critical diseases in multi-system organs (3). Some studies believe that by attaching importance to the role of conceptual framework, it will help to further clarify and promote the teaching objectives and oriented education in ICU, and therefore, clinical skills training based on simulation technology and intelligent tools is generally adopted in ICU teaching at present (4). In addition, some studies have found that the teaching style and supervision level of clinical teachers in intensive medicine are closely related to the learning behavior and training effectiveness of residents. By understanding the learning behaviors and expectations of residents trained in ICU, and appropriately adding clinical skills training supplemented by appropriate teaching methods and supervision levels, it will be beneficial and win-win for both ICU department construction and clinician training (5, 6). The above indicates that it is of great practical significance for higher medical educators to explore timely and effective clinical teaching strategies of intensive medicine.

BOPPPS is a novel clinical teaching model, which stimulates resident physicians’ learning interest through bridge-in (B) introduction, and draws up corresponding training objectives (O) and pre-assessment (P) plans according to the actual situation of students. Various teaching methods are integrated to allow each resident physicians to participate in learning (P), and post-assessment (P) is used to evaluate the resident physicians’ mastery of knowledge and skills and make a summary (S) (7, 8). Although BOPPPS is generally helpful for clinical teachers to organize the teaching process clearly and flexibly and stimulate students’ learning initiative, the applicability and practical significance of BOPPPS model in modern medical education are largely restricted due to the limitation of time and space in offline or bedside teaching which is especially evident in the stages of assessment, summary and feedback (9). Consequently, it is urgent to consider the optimization of BOPPPS teaching method and combine with other teaching modes.

Situational teaching method is a teaching approach that employs modern diversified technological models to simulate real clinical practice scenarios of patients during the conduct of clinical operations and learning. It has gradually emerged as another major direction of medical education reform, boasting strong repeatability and high safety. Situational teaching method takes the lead in simulating and designing diagnosis and treatment scenes related to clinical teaching contents and objectives in the teaching process, and then vividly show the complexity and diversity of clinical diseases in ICU, so as to stimulate and enhance the learning interest and motivation of trainees (10, 11). There is no denying that situational teaching method offers convenience and a close-to-real environment for clinical teaching practice, averting the ethical issues resulting from relying on actual patients for teaching and providing an excellent method and means for medical teaching.

Accordingly, combining advantages of BOPPPS with benefits of the situational teaching method may be likely to gain some benefits in ICU clinical teaching practice. As the key clinical specialty of intensive medicine in our medical centers from Fujian and Jiangxi province of China, we had carried out the clinical teaching practice exploration by combining BOPPPS with situational teaching method in recent years, and achieved commendable teaching practice results, which are summarized and shared in this article.

## 2 Materials and methods

### 2.1 Study design and settings

This randomized trial was conducted using a multi-center, prospective cohort study in accordance with the ethical principles of medical research in the Declaration of Helsinki, and this study has been reviewed and approved by the Medical Ethics Committee of Fujian Medical University Union Hospital, the Affiliated Hospital of Putian University and the First Affiliated Hospital of Gannan Medical University. In this study, Epi-info software was utilized to determine the sample size. A total of 293 residential physicians training in the ICU of Fujian Medical University Union Hospital, the Affiliated Hospital of Putian University and the First Affiliated Hospital of Gannan Medical University from January 2021 to December 2023 were selected as the study subjects, and according to the random number table method, 142 residential physicians were randomly divided into the control group and 151 residential physicians in the experimental group. The whole process of teaching method in this study is voluntary and anonymous.

### 2.2 Autonomous evaluation method

This study adopts a hybrid approach, using quantitative experiments and descriptive qualitative methods in complementary design. Qualitative findings were used to provide further insight and explanation for quantitative results. For the purposes of the study, the observational measures were structured around the teaching/learning process, outcomes (achievement scores) and personal feelings (teaching satisfaction).

### 2.3 Inclusion and exclusion criteria

Inclusion criteria: (1) Resident physicians who participating in the standardized training in ICU; (2) Those who have certain clinical professional skills and good language expression ability and organization and coordination ability, accept guidance and compliance is applicable.

Exclusion criteria: (1) Those who did not complete the standardized training task of intensive medicine residents and quit midway; (2) Those who participate in this teaching research but lack relevant data; (3) There is no guarantee that learning or training will be conducted in strict accordance with the pedagogy involved in this study; (4) Participating in other teaching training or teaching researchers in our institute. The subjects included in the study all agreed to this study and volunteered to participate in this teaching research.

### 2.4 Research methods

At the initial stage of the implementation of this study, we accomplished the recruitment and training of clinical teachers in each medical center, and to ensure that the research process and standardization can be homogenized. Through the random number table method, 8–12 residential physicians who came to ICU for rotation training each month were divided into the control group and the experimental group, ensuring that the number of residents in the two groups was as equal as possible. And it was mandatory to ensure that the clinical teaching and training content received by the residents in the two different teaching groups was consistent, but the training methods and modes (pedagogies) received by the residents in the two groups were significantly different. The control group adopted the traditional teaching and demonstration teaching method, and experimental group adopted the situational teaching method guided by the BOPPPS model. All kinds of critical and severe diseases admitted in ICU and ICU routine operation skills were integrated into the daily training and graduation assessment as training items. It should be noted that the curriculum system and training of ICU clinical teaching encompass the following 10 modules:

Module 1. Cardiopulmonary resuscitation + Use of simple respirator + Tracheal intubation + Electrocardioversion.
Module 2. Ventilator operation and alarm handling.
Module 3. Deep venous catheterization + Monitoring of central venous pressure.
Module 4. Arterial puncture catheterization (invasive blood pressure monitoring).
Module 5. Fiberbronchoscopy + Alveolar lavage.
Module 6. Percutaneous tracheotomy.
Module 7. Blood purification technology.
Module 8. Aortic balloon counterpulsation technique.
Module 9. PICCO monitoring technology.
Module 10. Temporary pacemaker implantation.

**Figure 1:**
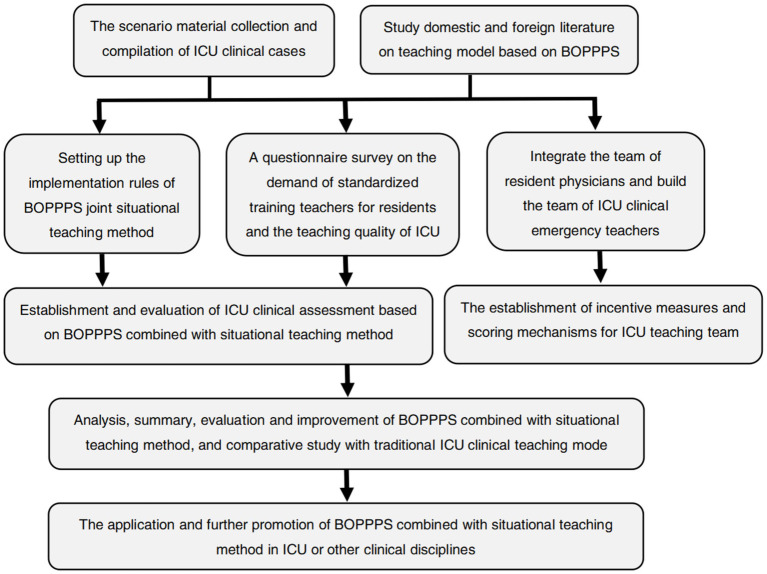
The practice application model diagram of BOPPPS model combined with situational teaching method in clinical training of intensive medicine.

The above 10 distinct clinical teaching modules are clinically instructed by 10 clinical teachers recruited and selected by each medical center, and it is ensured that the clinical teaching content of the control group and the experimental group during the ICU training is the content of the 10 teaching modules, yet the teaching approaches and models adopted are different. The clinical training content of this study takes module 3 “Deep venipentesis catheteization + Monitoring of central venous pressure” as an instance. The training cycle is 3–5 residential physicians per team, and the teaching duration is no more than 2.5 h (including the time for independent training and integrated skills). The teaching practice process of this study is shown in Figure 1 “The practice application model diagram of BOPPPS model combined with situational teaching method in clinical training of intensive medicine.”

### 2.5 Clinical teaching method in control group

The control group adopted the traditional bedside teaching, explanation and demonstration of ICU skills operation mode, specifically: First, the teacher explained the operation process, points of attention and relevant knowledge points of “Skills operation and scoring criteria for deep vein puncture and catheterization of ICU,” so that each student had a preliminary understanding and understanding of this operation training; Secondly, after the teaching and explanation is completed, multimedia skill operation videos can be used to simulate patients on site. After that, simulate patients (SPs) can be used to simulate the operation. Then let the trainees ask questions one by one, answer by themselves, and then decompose the action and demonstrate the standard for the difficult, key and error-prone points of the operation, and the trainees watch next to them; Finally, the students trained independently, the teachers conduct inspection and answer questions.

### 2.6 Clinical teaching method in experimental group

The experimental group adopted the BOPPPS model combined with situational teaching method, that is, a new teaching mode guided by clinical teaching goal and centered on students, actively creating specific clinical ICU diagnosis and treatment or first aid situations. The BOPPPS model consists of six links: teaching introduction (bridge), teaching objectives, pre-assessment, participatory learning, post-assessment and summary. It should be noted that situational teaching method can be applied to SPs, PPT, virtual reality and other multimedia technologies to carry out, and the whole process integrates the situational teaching method into each step of the BOPPPS model. The training period of this new pedagogy in the ICU typically does not exceed 2.5 h, and the key information, interventions, and evaluation tools for the implementation of the BOPPPS model are as follows:

(1) Teaching introduction (Bridge, within 15 min): at the beginning of clinical teaching, the clinical teacher in charge of this module will introduce the situation of vivid and interesting clinical cases, problem analysis, skill operation videos and new progress of diagnosis and treatment. In order to attract students’ attention and interest, the introduction method pays attention to appropriate methods and techniques, and pays attention to the lively and interesting nature. At the same time, the introduction should be as concise as possible, focusing on the effective connection between this training content and students’ existing theoretical knowledge of ICU or problems that may be encountered in future clinical practice. In this part, we attached importance to the theme of situational teaching method and use information technology and multimedia technology to create a vivid simulated clinical situation.
(2) Teaching objective (within 15 min): the purpose of this stage is to make residential physicians clear about the learning objectives of ICU rotation, so as to facilitate them to grasp the focus of ICU clinical knowledge. Teaching objectives include knowledge, literacy and skills. The objectives should be set from the perspective of training students. The objectives should be clear (what clinical knowledge points need to be mastered), appropriate (related to the topic of ICU), accessible (within the scope of students’ ability), and measurable (setting evaluation indicators).
(3) Pre-assessment (within 20 min): the purpose of pre-assessment is to master the training ability of rotating residential physicians, understand their interest in ICU clinical knowledge and skills and prior knowledge, so as to facilitate the subsequent adjustment of the depth and progress of clinical teaching, and make clinical teaching objectives more focused. It can usually be conducted in the form of quizzes, questions and answers, or group discussions. The pre-assessment questions set in this part are closely related to the content background and basic theoretical knowledge of clinical training. The amount of questions is controlled within 4 questions, and the pre-assessment contents of 10 modules in various medical centers in this study are consistent, so as to facilitate measurement and comparison and minimize the interference of human factors.
(4) Participatory learning (within 50 min): focusing on the idea of “students as the main body.” After teaching the concepts, key points and difficulties of the knowledge points of a clinical situation in ICU, rich and interesting forms such as personal report, group discussion, clinical practice, role play, special discussion and case analysis can be used to fully stimulate the learning enthusiasm of residential physicians and guide students to actively participate in the clinical teaching and training of ICU. In order to deepen the understanding of the training content, it also strengthens the cultivation of young doctors’ doctor-patient communication ability, teamwork ability, clinical skills and other qualities. Of course, in this section, it is essential to guarantee that clinical teacher assume the role of participants and contributors, while residential physicians serve as the “leaders” of this section. It is indispensable to enable each resident to engage in simulated clinical scenarios, to investigate and solve problems in clinical practice, and to effectively execute clinical skills.
(5) Post-assessment (within 20 min): post-assessment can also be understood as a course assessment, which aims to judge whether residential physicians have achieved the expected results of ICU clinical teaching. It is required to evaluate the clinical teaching effect in the course assessment or teaching process, and the clinical teaching effect can be evaluated through the examination questions, clinical questionnaires, skill assessment, special reports and other ways, and according to the evaluation results, clinical teaching reflection and rectification, timely adjustment of teaching design and teaching content form, so as to better achieve the teaching objectives of ICU. In this section, we suggest that it should contain no more than 5 questions, which can be overlaps with the few contents previously tested, so as to consolidate the knowledge of clinical medicine. Of course, it is more important to ensure that the clinical training content can be extended to clinical practice, so that each residential physicians can master the core of this training.
(6) Summary (within 15~25 min): To further deepen the impression of students by summarizing the key points of clinical knowledge and the context of skill assessment during ICU training. Different from the traditional teaching mode, BOPPPS combined with scenario simulation teaching emphasizes that residential physicians should summarize, summarize and reflect on their own clinical knowledge and skills. In the summary process, the young resident doctors should be the main body, and the clinical superior doctors should be the guidance role, so that each resident physician can summarize, share and supplement, and then the clinical superior doctors will emphasize the key points and difficulties.

Consequently, the BOPPPS model and the steps of the combined situational teaching method are applied to the clinical teaching practice of ICU, while guaranteeing that the entire teaching process does not exceed 2.5 h. In this study, the column of clinical teaching course design and implementation of “Deep venocentesis catheterization + Monitoring skills Operation and scoring Standards of central venous pressure” is taken as an example. The specific implementation steps, time frame, teaching process and intervention strategy can be detailed in Table 1.

**Table 1:** Skills operation and scoring criteria for monitoring central venous pressure in ICU (BOPPPS model combined with situational teaching version).

Procedure	Method and significance	Teaching implementation and technical route	Time (min)
Bridge	Set up vivid situations, introduce clinical cases, stimulate students’ interest in learning, and refine training objectives	A 25-year-old patient was admitted to the emergency department due to pelvic fracture and hemorrhagic shock caused by a car accident, and was referred to our department for physical examination: blood pressure 60/40 mmHg, heart rate 130 beats/min, SPO_2_ 98%. What is the first aid that the doctor should do before doing clinical treatment?	15 min
Objective	List at least 3 operational objectives and learning objectives, so that the trainees can understand the purpose of training and the significance of clinical rescue work in the future	Determine the patient’s consciousness and evaluate vital signs; Clean up respiratory secretions, maintain airway patency, and avoid pulmonary complications; Perform sputum aspiration as soon as possible to ensure smooth breathing and avoid asphyxia.	15 min
Pre- assessment	Design and order at least 3 questions to examine the students’ knowledge reserve and skill level	What are the common causes of hemorrhagic shock? What are the indications for deep vein puncture and catheterization? What are the complications of deep vein puncture and catheterization?	20 min
Participatory Learning	Senior doctors teach and train through explanation, demonstration, guidance, question-and-answer, etc. Students consolidate and improve the skills of “deep vein puncture and catheterization” by means of operation practice and skill simulation	Preoperative preparation Preparation of materials (demonstration + training simulation): stethoscope, disposable sterile kit (containing sterile operating gown + sterile towel + sterile towel), disposable sterile gloves, saline, heparin sodium 1, 2% glucochlorhexidine skin disinfectant, 2% lidocaine, central vein puncture kit, treatment cart, waste tank Personal preparation (demonstration + training simulation): check the identity of the patient, obtain the consent of the family and sign; Clean environment; Prepare the materials and check the date of production; Assess and monitor patient’s vital signs and consciousness, auscultate respiratory sounds in both lungs, and adjust ventilator PEEP level to 0 if necessary; Position preparation: pillow supine, head 15° ~ 30° lower, shoulder cushion, exposed neck, turn the head to the opposite side of the operator; Bedside ultrasound was used to probe the position of internal jugular vein for anatomic variation. Specific operation steps Aseptic operation (standard hand washing, wearing masks, hats, gloves); Disinfection and towel covering of the operative area; Check the quality of puncture supplies and pre-fill catheters with heparin water; Confirm the puncture site again: the tip of the sternocleidomastoid triangle is used as the puncture point, about the level of cricoid cartilage and the lateral side of carotid artery; The needle is at a 45° angle to the skin, pointing directly to the ipsilateral papilla; Local infiltration anesthesia; Venipunction, make sure the needle tip is in the central vein; Insert guide wire and insert the expander along the guide wire, make sure the catheter to a depth of 12 ~ 13 cm; Flush catheter with heparin water and catheter fixation; Post-operative treatment (instrument treatment, sharp instrument treatment, medical waste treatment); Wash your hands regularly and listen to the breathing sounds of both lungs again; Medical advice creation, writing records; Imaging confirms catheter depth (catheter tip is located near right atrium of superior vena cava); Young trained doctors practice in small groups to ensure that each student operates independently for 2 to 3 times. Other students present observe mistakes, and the training teachers inspect the training situation and answer questions in time.	50 min
Post-assessment	Through operation exercises, systematic question answering, practical training simulation, consolidation and improvement and other links, evaluate and assess whether each student has a solid grasp of deep vein puncture and catheterization	About 60% of the trainees in each group were selected for operation assessment, and other trainees watched quietly. After the operation was completed, the remaining 40% of the trainees who did not get the selection made comments on the problems in the operation process one by one, and finally the trainers made comments.	20 min
Summary	Review the training objectives, precautions, key points and operation procedures in the situational cases, summarize the practical training skills, and deepen the value of the training skills in clinical work	Wear a mask, medical hat and gloves before operation. Wash hands strictly and disinfect the operative area. Strict aseptic operation. The position of central vein was investigated by bedside ultrasound for anatomic variation. Avoid multiple piercings (≥3). Avoid puncture of arteries by mistake. Avoid false paths. Both lung respiratory sounds were ausculted before and after puncture.	20 min

### 2.7 Implementation of the intervention

In accordance with the requirements and arrangements of the practical training in critical care medicine, the different teaching modules are carried out by 10 teachers. They strictly adhere to the clinical teaching syllabus and curriculum standards to implement the clinical practice teaching lesson plan, minimizing the impact of previous clinical experience, basic knowledge, learning style, or cognitive ability on the training results. Certainly, throughout the teaching practice, teachers can contact members of the academic research team at any time if they have inquiries, and it is crucial to maintain the execution log and teaching records to document any deviation from the teaching plan and clinical practice training. Generally, this study is founded on the exploration and practice of the BOPPPS model combined with the situational teaching method in the clinical teaching of critical care medicine. There might be some deviations of diverse natures. However, to prevent such circumstances, all clinical teachers in the experimental group received the same teaching materials and resources from the research group, namely, student rosters, clinical practice teaching curriculum standards, BOPPPS practice worksheets, solution guides, explanatory videos, and multimedia materials for anchoring scenario simulation teaching to guarantee the homogeneous development and training of students in the experimental group at different stages of teaching and avoid mistakes.

### 2.8 Evaluation of teaching effect

Effective evaluation and improvement of the theoretical knowledge and clinical skills of the ICU team may help to improve compliance with clinical treatment goals and contribute to patient rehabilitation (12, 13). The clinical teaching results in this study were composed of three parts: ICU theoretical knowledge assessment, ICU clinical thinking and skills assessment and clinical critical case management assessment. Both groups of residential physicians were evaluated on Friday of the last week after the training of ICU. The examination items were all in the percentage system, including ICU theoretical knowledge, medical history collection, physical examination, medical record writing, discussion and analysis of medical examination results, ICU operation skills and rescue skills. Among them, the assessment of ICU theoretical knowledge is based on the “Medical theoretical test paper of ICU (A/B paper)” which is independently ordered by my department in accordance with the latest edition of the teaching syllabus, and the higher the score, the better the theoretical performance. Besides, the assessment of ICU clinical thinking and skills and the assessment of clinical critical cases are the focus of ICU clinical thinking and skills assessment. By sorting out the quantitative question Bank of ICU clinical Skills Operation Assessment, each student is allowed to select 2 to 3 items for assessment and evaluation. To examine the degree of mastery of common clinical skills and rescue techniques in ICU training.

To acquire a more lucid comprehension of the training column and the overall time planning of this study, as previously stated, the clinical teaching content encompassed in this study amounts to a total of 10 modules. Moreover, this study has consistently emphasized the homogeneity of the teaching research conducted in the three medical centers. Fortunately, each of our medical centers allocates 2 months (8 weeks) as the training cycle for a group of residential physicians. That is to say, we are required to complete 10 modules of clinical teaching content for a specific group of residents within 2 months. Additionally, for the purpose of testing and evaluating the outcomes and conducting the teaching satisfaction survey of the control group and the experimental group, we schedule the graduation assessment and teaching satisfaction survey on Friday of the last week (week 8). Our specific clinical training arrangements are presented in Table 2.

**Table 2:** The specific clinical training arrangement and time schedule planning of this study.

Module	Week	Clinical teaching (training) content	Training duration
1	1st, Thursday	Cardiopulmonary resuscitation + Use of simple respirator + Tracheal intubation + Electrocardioversion	<150 min
2	2nd, Thursday	Ventilator operation and alarm handling	<150 min
3	3rd, Thursday	Deep venous catheterization + Monitoring of central venous pressure	<150 min
4	4th, Thursday	Arterial puncture catheterization (invasive blood pressure monitoring)	<150 min
5	5th, Thursday	Fiberbronchoscopy + Alveolar lavage	<150 min
6	6th, Thursday	Percutaneous tracheotomy	<150 min
7	6th, Friday	Blood purification technology	<150 min
8	7th, Thursday	Aortic balloon counterpulsation technique	<150 min
9	7th, Friday	PICCO monitoring technology	<150 min
10	8th, Thursday	Temporary pacemaker implantation	<150 min
Additional bonus	8th, Friday	Clinical rotation assessment (including medical theory, clinical practice skills, comprehensive quality, etc.) and teaching satisfaction survey	<180 min

### 2.9 Evaluation of learning motivation

Learning motivation and critical thinking in clinical teaching are regarded as “comprehensive quality.” Improving clinical learning motivation and cultivating critical thinking ability is conducive to improving teaching quality and clinical professional level (14, 15). Among them, study process questionnaire (SPQ) is used to evaluate learning motivation, which mainly includes surface motivation, deep motivation and achievement motivation, with a total of 24 items. Likert 5-level scoring method was used for evaluation. The higher the score, the stronger the learning motivation. SPQ is an effective and valid instrument to measure the learning methods and motivation of residential physicians (16, 17). Therefore, SPQ was used to evaluate the learning motivation in this study.

### 2.10 Evaluation of critical thinking ability

Critical thinking (CT) is the basic quality and ability of medical students, nurses or young physicians. The Critical Thinking Disposition Inventory-Chinese Version (CTDI-CV) is often used to evaluate the critical thinking ability of medical students or residents after clinical teaching and training (18, 19). As for the evaluation of critical thinking ability in this study, the CTDI-CV is used to evaluate the critical thinking ability of the two groups of residential physicians, which mainly includes 7 dimensions. A total of 70 projects. Each item is scored from 0 to 5, with higher scores indicating stronger critical thinking.

### 2.11 Teaching satisfaction survey

Clinical teaching effectiveness needs objective feedback and evaluation from trainees. Teaching satisfaction survey is regarded as an indispensable feedback path in clinical teaching, which is helpful to improve the quality of clinical teaching. At present, many researches on medical teaching and reform have included teaching satisfaction survey as a key part of teaching research, and most of them are self-developed and designed questionnaires (20, 21). The effect evaluation and satisfaction questionnaire of ICU training (EESQ) with a total of 10 items was issued to the two groups of resident physicians in an anonymous manner. Its content generally includes eight dimensions, such as training objectives and learning interests, training atmosphere, improving humanistic care and doctor-patient communication ability, improving team cooperation ability, improving job identity, ICU rotation harvest and satisfaction with teacher style. The questionnaire options are divided into three gradients of “very satisfied, satisfied and dissatisfied,” satisfaction = (number of very satisfied cases + number of satisfied cases)/total number of cases ×100%. The resident students are evaluated objectively and anonymously throughout the whole process, without external interference such as human factors, and timely recovery and statistical analysis.

### 2.12 Statistical analysis

In this study, the SPSS 20.0 (IBM, New York, United States) was used to perform the analytic processes. The categorical variables were presented as number (percentage), while continuous variables were represented as mean (standard deviation) or median (interquartile range). Student’s *t* test was employed for group means when variables exhibited a normal distribution and homogeneity of variance. Chi-Square test or Fisher’s exact test was employed for group medians for categorical variables. Statistical significance was set as a two-sided *p* < 0.05.

## 3 Results

### 3.1 Demographic characteristics

A total of 293 residential physicians training in the ICU of three different medical centers (hospitals) from January 2021 to December 2023 were selected as the study subjects. Table 3 shows details on demographic characteristics of the study participants, and there was no statistical significance (all *p* > 0.05), which was comparable.

**Table 3:** Participant demographics.

Indexes	Control group (*n* = 142)	Experimental group (*n* = 151)	χ^2^*/t*	*p*
Gender [*n* (%)]			χ^2^ = 0.633	0.426
Male	63 (44.37)	74 (49.01)		
Female	79 (55.63)	77 (50.99)		
Age (year)	25.52 ± 2.47	26.09 ± 2.83	*t* = 1.832	0.068
Specialty of residents			*χ*^2^ = 1.384	0.967
ICU (Intensive medicine)	47 (33.09)	52 (34.44)		
Internal medicine	31 (21.83)	34 (22.52)		
Anesthesiology	19 (13.38)	20 (13.25)		
Surgery	28 (19.72)	31 (20.53)		
Oncology	7 (4.93)	5 (3.31)		
Obstetrics and pediatrics	6 (4.23)	7 (4.64)		
Other clinical majors	4 (2.82)	2 (1.32)		
Grade or level of residency			*χ*^2^ = 3.390	0.184
1	58 (40.84)	66 (43.71)		
2	49 (34.51)	38 (25.17)		
3	35 (24.65)	47 (31.12)		
Pre-training test scores	82.96 ± 5.78	84.23 ± 6.24	*t* = 1.804	0.072
Have the doctor qualification certificate [*n* (%)]			*χ*^2^ = 2.214	0.137
Yes	88 (61.97)	106 (70.20)		
No	54 (38.03)	45 (29.80)		
Educational background [*n* (%)]			*χ*^2^ = 2.681	0.102
Bachelor	95 (66.90)	87 (57.62)		
Master	47 (33.10)	64 (42.38)		

### 3.2 Comparison of clinical teaching results

As shown in Table 4, the scores of the experimental group in ICU theoretical knowledge, clinical thinking and skills assessment and the management of clinical critical cases were significantly better than those of the control group, with statistical significance (all *p* < 0.05).

**Table 4:** Comparison of assessment results between the two groups [(^−^x ± s), scores].

Assessment item	Control group (*n* = 142)	Experimental group (*n* = 151)	*t* value	*p* value
ICU theoretical knowledge	83.94 ± 12.73	87.31 ± 13.15	2.226	0.027
ICU clinical thinking and skills	88.37 ± 12.61	92.86 ± 12.35	3.079	0.002
Ability to deal with clinical critical cases	78.83 ± 10.47	81.45 ± 11.28	2.057	0.041

### 3.3 Comparison of SPQ scores of learning motivation

As shown in Table 5, The surface motivation, deep motivation, achievement motivation and SPQ total scores of the experimental group were all higher than those of the control group, and the differences were statistically significant (all *p* < 0.05).

**Table 5:** Comparison of SPQ scores between the two groups [(^−^x ± s), scores].

Assessment item	Control group (*n* = 142)	Experimental group (*n* = 151)	*t* value	*p* value
Surface motivation	29.78 ± 4.12	31.14 ± 4.69	2.630	0.009
Deep motivation	18.73 ± 2.34	20.56 ± 2.91	5.949	<0.001
Achievement motivation	29.53 ± 4.16	31.09 ± 4.53	3.065	0.002
Total scores	78.04 ± 5.73	82.79 ± 6.98	6.382	<0.001

### 3.4 Comparison of CTDI scores for critical thinking

As shown in Table 6, The scores of seek truth, open mind, analytical ability, systematic ability, self-confidence of critical thinking and intellectual curiosity of the experimental group were higher than those of the control group, and the CTDI total score was statistically significant (*p* < 0.05).

**Table 6:** Comparison of CTDI scores between the two groups [(^−^x ± s), scores].

Evaluation item	Experimental group (*n* = 151)	Control group (*n* = 142)	*t* value	*p* value
Seek truth	38.98 ± 3.12	37.75 ± 4.04	2.904	0.004
Open mind	43.23 ± 4.97	39.14 ± 5.21	6.877	<0.001
Analytical ability	40.87 ± 4.53	37.26 ± 4.15	7.099	<0.001
Systematic ability	40.05 ± 6.74	38.08 ± 5.09	2.834	0.005
Self-confidence of Critical thinking	40.28 ± 4.02	36.99 ± 4.83	6.316	<0.001
Intellectual curiosity	38.61 ± 4.97	34.94 ± 4.36	6.702	<0.001
Cognitive maturity	38.05 ± 3.75	37.23 ± 3.67	1.890	0.060
Total scores	280.07 ± 30.23	261.59 ± 31.45	5.128	<0.001

### 3.5 Teaching satisfaction questionnaire survey

As shown in Table 7, The “Satisfaction Questionnaire of ICU training” was issued in secret form, and 109 valid questionnaires were collected and statistically analyzed by group. The experimental group was superior to the control group in terms of learning interest in ICU, enhancement of humanistic care and doctor-patient communication ability, enhancement of team cooperation ability, enhancement of job identity, ICU training harvest and satisfaction with teacher style (all *p* < 0.05).

**Table 7:** Results of teaching satisfaction questionnaire of ICU [*n* (%)].

Evaluation item	Experimental group (*n* = 151)	Control group (*n* = 142)	*χ^2^* value	*p* value
Clear training objectives	133 (88.08)	115 (80.99)	2.833	0.092
Increase interest in ICU learning	139 (92.05)	109 (76.76)	13.165	<0.001
Active training atmosphere	127 (84.11)	111 (78.17)	1.692	0.193
Enhancement of humanistic care and doctor-patient communication ability	131 (86.75)	101 (71.13)	10.843	0.001
Enhancement of team Cooperation ability	134 (88.74)	103 (72.54)	12.433	<0.001
Enhancement of job identity	121 (80.13)	95 (66.90)	6.613	0.010
ICU training harvest	138 (91.39)	112 (78.87)	9.157	0.002
Satisfaction with teacher style	144 (95.36)	119 (83.80)	10.643	0.001

## 4 Discussion

As a closed-loop feedback course design model that focuses on teaching interaction and teaching reflection, BOPPPS is one of the most effective and attractive practice models for clinicians in the process of teaching design and clinical teaching (22). BOPPPS emphasized the all-round participation and interactive feedback between clinical teachers and students to promote trainees to better participate in clinical teaching activities, which can stimulate residential physicians’ learning enthusiasm and initiative, and give full play to the guiding role of clinical teachers (7). By simulating real medical circumstances, situational teaching method furnishes trainees with a learning setting similar to actual clinical operations. Such a clinical teaching method not only enables young residents to practice and enhance their skills in a secure environment but also boosts their proficiency and self-confidence in operational skills. The utilization of immediate feedback presents residents with valuable learning opportunities, assists them in identifying and correcting errors, and further enhances their clinical practice skills and overall competencies.

Of course, on the basis of the application of BOPPPS teaching mode, the combination of situational teaching method, according to the conditions of the time and local conditions, without adhering to the fixed theoretical knowledge of ICU, can better reflect the complexity and urgency of the daily practical diagnosis and treatment activities in ICU. Compared with the traditional clinical teaching method, BOPPPS model combined with situational teaching method can better stimulate the training enthusiasm and thirst for knowledge of ICU residents in different stages and processes of clinical teaching by implementing the teaching process of daily ICU diagnosis and treatment activities and skill items in steps. For example, with the help of the “Bridge-in (introduction)” part, the ICU clinical situation cases are vividly described and discussed, and the “pre-assessment” is used to deepen the students’ cognition of the sudden, complexity and difficulty of the ICU clinical work, which better stimulates the students’ desire to strengthen their own skills and effectively mobilizes the subjective initiative. While In the “post-assessment” link, the teacher can randomly select the trainees for demonstration and evaluation, enhance the interaction between teachers and students and students, enhance their subjective initiative and enhance the learning motivation of students, extremely effectively improve the quality and teaching effect of ICU clinical teaching.

Generally speaking, the daily clinical teaching practice of ICU is mostly taught by senior and experienced associate chief physicians, often including “explanation,” “demonstration,” “independent practice” and “summary” and other steps. However, due to the heavy work of daily diagnosis and treatment in ICU, the residents lack effective supervision in the process of independent training, and some residents are prone to be lazy and unwilling to operate. The lack of innovation and flexibility is not conducive to improving the training of team cooperation ability of young residents. By introducing the BOPPPS model combined with situational teaching method into ICU clinical teaching, residents are “double supervised” by peers and teachers in the training process, and in the process of “participatory learning,” the same group of residents are more cohesive, and the team cooperation ability and critical thinking are better cultivated. It is also conducive to the cultivation of team consciousness and the construction of team spirit in the future complicated clinical diagnosis and rescue work, which is conducive to the harmony between doctors and patients in the long run.

Through the evaluation of ICU training effect and the investigation and analysis of teaching satisfaction of the two groups included in this study, we were surprised to find that compared with the traditional clinical teaching mode, the experimental group using the BOPPPS model combined with situational teaching method was significantly better than the control group in terms of ICU theoretical knowledge, operational skills, humanistic care and doctor-patient communication. Moreover, the evaluation and satisfaction of the training effect of the experimental group were also higher than that of the control group, indicating that the introduction of the BOPPPS model combined with situational teaching method into the clinical teaching practice of ICU not only correlated the design of clinical teaching links with the daily diagnosis and treatment activities of ICU, but also had strong operability and good effect in the actual clinical teaching process of ICU. In addition, it can improve the satisfaction of residential physicians for learning in ICU training, and improve the teaching enthusiasm and enthusiasm of ICU clinical teachers to a certain extent, so as to achieve the goals and tasks of ICU clinical teaching and training.

The clinical work in ICU is complicated, laborious and risky, and requires high knowledge reserve and clinical skills of residential physicians (23, 24). To this end, the BOPPPS model combined with situational teaching method is introduced into the clinical teaching practice of ICU, and young residents are allowed to think from the perspective of patients and their families through some fresh case scenarios, helping them to establish a good sense of professional identity and responsibility. And in the ICU skills training process to master and consolidate knowledge and skills, enhance the spirit of saving the dead and healing the wounded, experience their own responsibilities as an angel in white and other aspects have played a good role in promoting. Correspondingly, the “participatory learning” and “post-assessment” links in the BOPPPS model of the combined situational teaching method could enable resident physicians to experience the team friendship of partners who enter ICU training at the same time, deepen their feelings and identification with the hospital, and at the same time, through certain evaluation and demonstration means in the “summary” link. In the process of interaction, exercise, teaching and thinking, the residential physicians’ sense of belonging to the unit is constantly enhanced, which is conducive to the career development of the resident physicians in the future clinical posts in the hospital.

As an important way for the growth of every young doctor, the standardized training of residential physicians is also the key to the training of young qualified doctors (25, 26). As ICU is a highly specialized clinical department that plays a special role, it is also an important reflection of the treatment level and core competitiveness of general hospitals. By introducing the BOPPPS model and situational teaching method into the clinical teaching practice of ICU, it improved the learning motivation and enthusiasm of resident physicians, but also effectively improve their doctor-patient communication ability, clinical comprehensive ability and rescue level, and help young doctors to establish good medical ethics and humanistic literacy, it also helped the resident physicians to develop good critical thinking.

## 5 Conclusion

Admittedly, we will persist in exploring the practice and application of the BOPPPS model in combination with the situational teaching method in clinical training for intensive medicine in the future. Additionally, by tracking or following up on whether there is any disparity in professional competence, clinical skill level, comprehensive ability, and scientific research level between the residential physicians of the experimental group and the control group who have undergone training in our medical center previously, longitudinal research will be conducted in the future, with the aim of exploring the long-term value and significance of the BOPPPS model combined with the situational teaching method in clinical teaching and training. Taken together, BOPPPS combined with situational teaching method may have significant practical effect in clinical teaching process of ICU and it will be a novel and valuable pedagogy in the clinical teaching of intensive medicine, which is worthy of reference and promotion in teaching hospitals.

## Data Availability

The original contributions presented in the study are included in the article/supplementary material, further inquiries can be directed to the corresponding author.
